# Doubly committed Subarterial Ventricular Septal defect repair: An experience of 51 cases

**DOI:** 10.12669/pjms.335.13429

**Published:** 2017

**Authors:** Tariq Waqar, Muhammad Farhan Ali Rizvi, Ahmad Raza Baig

**Affiliations:** 1 Tariq Waqar, FCPS, FRCS. Associate Professor of Pediatric Cardiac Surgery, CPE Institute of Cardiology, Multan, Pakistan; 2 Muhammad Farhan Ali Rizvi, FCPS. Senior Registrar Cardiac Surgery, CPE Institute of Cardiology, Multan, Pakistan; 3 Ahmad Raza Baig, B.Sc. Hons. CPT. Clinical Perfusionist, CPE Institute of Cardiology, Multan, Pakistan

**Keywords:** Aortic regurgitation, Doubly committed sub- arterial (DCSA), Ventricular septal defect

## Abstract

**Objective:**

To review the surgical outcome of doubly committed subarterial (DCSA) ventricular septal defect repair.

**Method:**

Data of 51 patients of doubly committed sub arterial Ventricular septal defect from January 2012 to June 2017 that were referred to Chaudhary Pervaiz Elahi institute of Cardiology Multan was retrospectively reviewed using electronic database. All patients were operated by first author. In our study, we segregated patients into four main groups depending on presence or absence of aortic structural defect and degree of aortic valve regurgitation. Group-A comprised of nineteen (37%) patients who neither had aortic structural nor functional abnormality while Group-B had six (11.7%) patients, having aortic valve cusp prolapse without aortic regurgitation. Group-C and Group-D consists of seventeen (33.3%) and nine (17.6%) patients respectively depending upon degree of aortic regurgitation. Furthermore, six (11.7%) patients among these 51 had associated defects as well.

**Results:**

Group-A patients had no aortic valve regurgitation post operatively and at follow up of 22.6 months (1.8 years) as well. In Group-B, aortic valve was not addressed and aortic regurgitation was also not present post operatively or on follow up of 33.6 months (2.8 years). Similarly, in Group-C aortic valve was not addressed, these patients also did not show progression of disease on regular follow up of 18 months (1.5 year). While, in Group-D, eight patients underwent aortic valve repair and one patient had aortic valve replacement. Aortic regurgitation improved significantly in all patients of this group and remained unchanged on follow up of 22.7 months (1.8 years).

**Conclusion:**

Early closure of doubly committed subarterial ventricular septal defect with appropriate management of aortic valve disease can halt the process of disease progression.

## INTROUCTION

Among various subtypes of ventricular septal defects, doubly committed subarterial (DCSA) type is of paramount importance. Firstly, because it has high prevalence in Asian population (30%) as compared to Western population (5-10%).^1^ Secondly, because of its association with aortic valve prolapse and aortic regurgitation(AR) which is estimated to exceed 40%.^2^

Aortic cusp prolapse and aortic regurgitation associated with DCSA is not a congenital lesion. It is an acquired condition and least expected to be diagnosed in infancy.^3^ Prolapse of right coronary cusp is more common than non-coronary cusp while left cusp is rarely involved. The underlying pathology is absence of muscular support which is given by attachment of sinus of Valsalva to the ventricular septum leading to prolapse of affected cusp and regurgitation.

Various hypothesis has been proposed to explain the association of aortic valve prolapse but most commonly accepted mechanism is Venturi effect. This presumes the vulnerability of aortic valve cusp to be drawn into nearby VSD as very high velocity blood shunts from left ventricle to low pressure right ventricle thus causing the cusp to prolapse.^4^,^5^

Aortic regurgitation develops after second year of life and becomes severe usually at age of twenty. Aneurysm of Valsalva is likely to develop in these patients and usually present in third or fourth decade of life.^6^

We used our data of DCSA patients to review our results as this is first ever study conducted on DCSA VSD patients in Pakistan.

## METHODS

From January 2012 to June 2017, 51 patients of DCSA were referred to us for surgical consultation. There was 36 male patients and 15 female patients. DCSA ventricular septal defects associated with Tetralogy of Fallot and other complex congenital defects were excluded from this study. Approval from department of Academic Affairs of CPE Institute of Cardiology, Multan was taken for this study. Late referral of patients and paucity of follow up were two most important reasons for which our institutions had the policy to get all patients of DCSA VSD admitted and operated as soon as they were referred to us thus avoiding future complications. The patients were diagnosed by routine 2-dimensional echocardiography and aortic regurgitation was graded by color Doppler echocardiography. Cardiac catheterization is not a routine for all patients unless pediatric cardiologist feels the need. VSD was categorized as large, moderate and small by comparing its size to aortic annulus diameter. Large VSD was 75% or greater of aortic annulus diameter, moderate 75%-33% of aortic annulus diameter and small was less than 33% of aortic annulus diameter. Aortic regurgitation was graded mild, moderate and severe on the basis of color flow Doppler.

For the purpose of description, patients were divided into four groups as shown in Table-I. Group-A comprised of 19 patients with no aortic regurgitation. Group-B consists of 6 patients with aortic valve structural abnormality in the form of cusp prolapse but without aortic regurgitation, so aortic valve was not addressed in these patients. In Group-C, 17 patients were included in whom aortic regurgitation was trace to mild and aortic valve was not addressed. Group-D constitutes of those 9 patients having moderate to severe aortic valve regurgitation therefore, aortic valve was repaired or replaced in these cases.

**Table-I:** DCSA VSD with Aortic Valve Physiology.

*Group*	*Variable*	*Frequency (Percentage)*
A	Normal Aortic Valve	19 (37.25%)
B	Cusp Prolapse without Aortic Regurgitation	06 (11.76%)
C	Aortic Regurgitation (trace to mild)	17 (33.33%)
D	Aortic Regurgitation (moderate to severe)	9 (17.6%)

Associated defects were present in 6 patients, rupture sinus of Valsalva in one patient, patent ductus arteriosus in 2, pulmonary stenosis in 2 and atrial septal defect in 1 patient. Bidirectional shunt was present in 3 patients, one patient had right to left shunt because of associated severe pulmonary stenosis, rest had left to right shunt.

SPSS v23 was used for statistical analysis. Quantitative variables were presented as mean value along with range. ANOVA test was used to compare quantitative operative and post-operative variables between the groups. F-ratio and p-value was calculated for all comparisons. P-value ≤0.05 was taken as statistically significant difference.

### Surgical Procedure

After median sternotomy, standard CPB was established after cannulating ascending aorta and venae cavae. Heart was arrested by antegrade cold blood cardioplegia. Intraoperatively, we assessed aortic insufficiency by arterial tracing while on bypass and aortic route filling and left ventricle distension while giving cardioplegia. In patients with moderate to severe aortic regurgitation, Aorta was opened and cardioplegia was given directly in coronaries by ostial cannula. Moderate hypothermia was achieved for myocardial protection. Ventricular septal defect was closed with dacron patch using interrupted pledgetted 5/0 prolene sutures. Some stitches were placed through the base of pulmonary leaflets to secure the patch to VSD. We used pulmonary approach in all cases except two, in whom VSD was closed by combined approach of right atrium and pulmonary artery. We did not repair aortic valve in cusp prolapse or mild degree of valve insufficiency. Aortic valve was repaired by suspension of right coronary cusp as described by Trusler in 8 patients. We reduced annulus with subcommissural stiches in one patient. In a five-year old boy with diagnosis of ruptured sinus of valsulva with deformed bicuspid aortic valve, we replaced it with 19mm Saint Jude metallic prosthesis. Transesophageal echocardiography was used to confirm adequacy of valve repair while coming of bypass. Associated defects like PDA, ASD and pulmonary stenosis were repaired during procedure.

## RESULTS

Postoperative Echocardiography revealed neither residual VSD nor significant pulmonary regurgitation. Complications included arrhythmias in 2 patients, pneumothorax in 1 patients, chest infection in 2 patients, all were managed successfully. There was no operative mortality. Operative and postoperative outcome variables shown in Table-II.

**Table-II:** Comparison of Operative and Post-Operative Outcomes.

	*Group-A VSD+ Normal AV*	*Group-B VSD+AV Cusp Prolapse*	*Group-C VSD+Trace to Mild AR*	*Group-D VSD+Moderate to Severe AR*	*F-Ratio*	*P-value*
Weight (Kg)	22.63 (11-50)	26.20 (10-64)	19.41 (10-51)	19.55 (11-28)	0.58	0.63
Bypass Time (min)	76.31 (53-100)	60.16 (46-69)	87.0 (67-121)	114.22 (89-229)	9.13	<0.001
Aortic Cross Clamp Time (min)	38.78 (28-66)	27.66 (21-35)	51.35 (28-88)	73.55 (54-180)	7.94	<0.001
Mechanical Ventilation Time (hours)	5.05 (3-9)	5.17 (3-10)	6.64 (4-11)	8.44 (6-20)	3.81	0.02
ICU Stay (hours)	27.15 (12-72)	29.83 (18-69)	23.88 (22-24)	26.88 (24-48)	0.47	0.70
Chest Drain (ml)	125.26 (30-730)	216.33 (84-790)	136.52 (45-510)	120.11 (75-250)	0.59	0.62
Hospital Stay (days)	5.57 (4-9)	5.0 (5-5)	5.76 (5-8)	5.44 (5-7)	0.78	0.51

### Group-A

Mean age of Group-A patients was 11.42 (3-21 years). VSD was small in 4 patients, moderate in 5 patients and large in 10 patients. Aortic regurgitation did not show up in any of these patients after mean follow up of 22.6 months (2.2 years).

### Group-B

Mean age was 9.83 years (2-17 years). VSD was small in 5 patients and large in single patient. Aortic valve was not addressed in these patients because they had only cusp prolapse without AR. VSD closure alone was found to be sufficient to halt Venturi effect. No patient showed progression of Aortic valve prolapse leading to regurgitation after mean follow up of 33 months (2.8 years).

### Group-C

Mean age was 10.33 years (3-18 years). Preoperative echocardiography of these patients showed trace to mild Aortic regurgitation and per operative evaluation confirmed the Echocardiography findings. So, aortic valve was not addressed in these patients. Postoperative Echo revealed no aortic regurgitation in 3 patients while it was same as preoperative echo in rest of the patients. Postoperative follow up did not show aortic valve disease progression after mean follow up of 18 months (1.5 years) in any patient.

### Group-D

Mean age was 10.76 years (4-19 years). VSD was small in 3, moderate in 5 and large in one patient. These patients had moderate to severe aortic valve disease. Aortic valve was repaired in 8 patients and replaced with 19 mm Saint Jude aortic valve metallic prosthesis in one patient. Aortic regurgitation was reduced to mild degree in 5 patients, mild to moderate in one patient while it vanished completely in two patients after repair. Single patient with aortic valve replacement showed adequately functioning aortic valve prosthesis with no para valvular leak and AVPG (Mean) 10mm Hg. No patient showed progression of Aortic regurgitation after mean follow-up of 22.7 months (1.8 years).

## DISCUSSION

DCSA accounts for approximately 1/3^rd^ (32%) of all surgically closed VSD^7^ in Asian population. While spontaneous closure of other types of VSDs is approximately between 25 to 50%^8^, Eugene and colleagues found that only 1 out of 128 patients with DCSA underwent spontaneous closure.^9^ DCSA is not a simple congenital anomaly, as it involves Aortic valve earlier in life. The association of aortic valve disease is estimated to be 40%.^2^ In contrary, our study revealed that Aortic valve disease was present in 62% (32 out of 51) of patients most likely due to the fact that our patients had late presentation, though need to repair was present only in 6 patients. In our study, not a single patient with DCSA involved Aortic valve before the age of 3 years as is suggested by other studies that aortic valve disease with DCSA VSD is an acquired lesion.^10^

The patients with most severe involvement of aortic valve disease in our study had mostly small VSDs. The association of small VSD with more severe aortic valve disease may explain Venturi effect as the predominant mechanism behind cusp prolapse. Smaller the defect is, higher will be the gradient across it and it will cause the blood to shunt across more vigorously thus drawing the nearby cusp in it and causing it to prolapse.

Patients with more severe disease in our study are of older age group. This indicates that once aortic valve disease develops, it is a progressive disease as showed by other studies.^11^

Some studies have observed that once AR develops, it will progress even after closure of VSD. It may end up in need of aortic valve replacement. De Leval and colleagues estimated 14%^12^, similarly, Chauvaud^13^ observed 16% need of aortic valve replacement even after VSD closure. Contrary to these studies, we found closure of VSD or aortic valve repair will halt the process of further damage to aortic valve. However, longer follow up studies and larger sample size are recommended to ascertain this fact.

The absolute timing for DCSA closure is not certain. In developed countries, where compliance of patients is good, some suggest that if degree of aortic valve prolapse is more than mild or progressing, then it should be operated. Nevertheless, most argue that any sign of inception of Aortic valve prolapse necessitates closure of DCSA VSD.^14^,^15^

Variety of procedures have been adopted for repair of prolapsing aortic valve cusps in DCSA patients. Among the various techniques, most widely practiced technique is that of Trusler and colleagues that was initially introduced by Carbol and later popularized by Trusler GA.^16^,^17^ Chauvad presented the comparison among the two groups of patients undergoing aortic valve repair by two different techniques. Group-A underwent plication of cusp and free edge folding while repair was accomplished by resecting the prolapsed portion of cusp and strengthening the aortic wall along with annuloplasty in Group-B patients. Former required reoperations only in 4% of cases while 43% of patients of later group had to undergo reoperations.^16^

Vigorous and progressive nature of aortic valve disease in DCSA, paucity of follow up in our society, late referral and poor compliance of patients are most important reasons which guide our policy to admit and operate the patient of DCSA as they are referred to us. We use Trusler repair for prolapsing aortic valve cusps. Usual approach is through the pulmonary artery but sometimes we had to open the right atrium when inferior edge of VSD is not clearly visualized. We recommend early closure of DCSA VSD because of progressive nature of disease. VSD closure in early stages suffices to halt the progression of aortic valve disease. Nevertheless, if aortic valve regurgitation has started it must be addressed to prevent future complications.

## CONCLUSION

Our experience suggests that doubly committed subarterial ventricular septal defect is not only more prevalent in our population (Asian) but also more associated with aortic valve insufficiency in comparison to western ethnic population. Early closure of ventricular septal defect with appropriate management of aortic valve disease can halt the process of disease progression.

### Author’s Contribution

**TW:** Conceived, designed the research methodology, prepared this manuscript and is accountable for the originality of the research work.

**MFAR and ARB:** Did data analysis, helped in writing the manuscript and reviewed the manuscript.
